# cGAS/STING: novel perspectives of the classic pathway

**DOI:** 10.1186/s43556-020-00006-z

**Published:** 2020-09-20

**Authors:** Menghui Gao, Yuchen He, Haosheng Tang, Xiangyu Chen, Shuang Liu, Yongguang Tao

**Affiliations:** 1 Key Laboratory of Carcinogenesis and Cancer Invasion, Ministry of Education, Department of Pathology, Xiangya Hospital, Central South University, Hunan, 410078 China; 2 NHC Key Laboratory of Carcinogenesis (Central South University), Cancer Research Institute and School of Basic Medicine, Central South University, Changsha, 410078 Hunan China; 3 Department of Oncology, Institute of Medical Sciences, National Clinical Research Center for Geriatric Disorders, Xiangya Hospital, Central South University, Changsha, 410008 Hunan China; 4 Hunan Key Laboratory of Tumor Models and Individualized Medicine, Department of Thoracic Surgery, Second Xiangya Hospital, Central South University, Changsha, 410011 China

**Keywords:** cGAS, STING, DNA sensor, Immune, Nuclei, Micronuclei, Tumor, Senescence

## Abstract

Cyclic GMP-AMP (cGAMP) synthase (cGAS) is a cytosolic DNA sensor and innate immune response initiator. Binding with exogenous or endogenous nucleic acids, cGAS activates its downstream adaptor, stimulator of interferon genes (STING). STING then triggers protective immune to enable the elimination of the pathogens and the clearance of cancerous cells. Apparently, aberrantly activated by self-DNA, cGAS/STING pathway is threatening to cause autoimmune and inflammatory diseases. The effects of cGAS/STING in defenses against infection and autoimmune diseases have been well studied, still it is worthwhile to discuss the roles of cGAS/STING pathway beyond the “classical” realm of innate immunity. Recent studies have revealed its involvement in non-canonical inflammasome formation, calcium hemostasis regulation, endoplasmic reticulum (ER) stress response, perception of leaking mitochondrial DNA (mtDNA), autophagy induction, cellular senescence and senescence-associated secretory phenotype (SASP) production, providing an exciting area for future exploration. Previous studies generally focused on the function of cGAS/STING pathway in cytoplasm and immune response. In this review, we summarize the latest research of this pathway on the regulation of other physiological process and STING independent reactions to DNA in micronuclei and nuclei. Together, these studies provide a new perspective of cGAS/STING pathway in human diseases.

## Introduction

Human body has a complicated defensive system against foreign pathogens, senescent and cancerous cells to maintain internal homeostasis. In this process, correctly detecting aberrant molecules is the first and foremost step, where two main immunity strategies – the adaptive immune system and the innate immune system – play indispensable roles. Adaptive immunity is performed by lymphocytes which are highly specific to a particular pathogen and provide long-lasting protection [1]. Unlike the adaptive immune system, the innate immune system is the first line of defense that respond to pathogens in a non-specific and generic way [2]. Extracellular pathogens are sensed and removed after binding to transmembrane receptors such as Toll-like receptors (TLRs), RIG-I-like receptors (RLRs) and NOD-like receptors (NLRs). When pathogens gain access into the cell or cell carcinogenesis happens due to harmful intrinsic damage, accumulated cytosolic DNA would function as a danger sign [3].

Cytosolic DNA delivers a signal of threat to innate immune system. Cytosolic DNA appears when certain pathogens infect cells or cellular genome is unstable. A number of mechanisms are involved in maintaining DNA level below the danger-signal threshold to prevent unnecessary waste of cellular energy. For example, the deoxyribonuclease (DNase) system. There are many types of DNases located in different subcellular sites. DNases located in the extracellular space (such as DNase I), endosomes (such as DNase II) and the cytoplasm (such as three prime repair exonuclease 1 (TREX1, also known as DNase III)) are all responsible for disposing mis-localized DNA [4]. But in some pathological conditions, abnormally distributed or accumulated DNA activates the self-defense mechanisms of cells by binding to DNA sensors. For example, in bacterial infection, unmethylated CpG DNA is recognized by TLR9 in endolysosomal compartment [5]. Absent in melanoma 2 (AIM2) detects DNA in the cytoplasm and activates the inflammasome pathway in response to exogenous and endogenous DNA challenge [6]. In some cases, RNA polymerase III also acts as a DNA sensor [7].

**Fig. 1:**
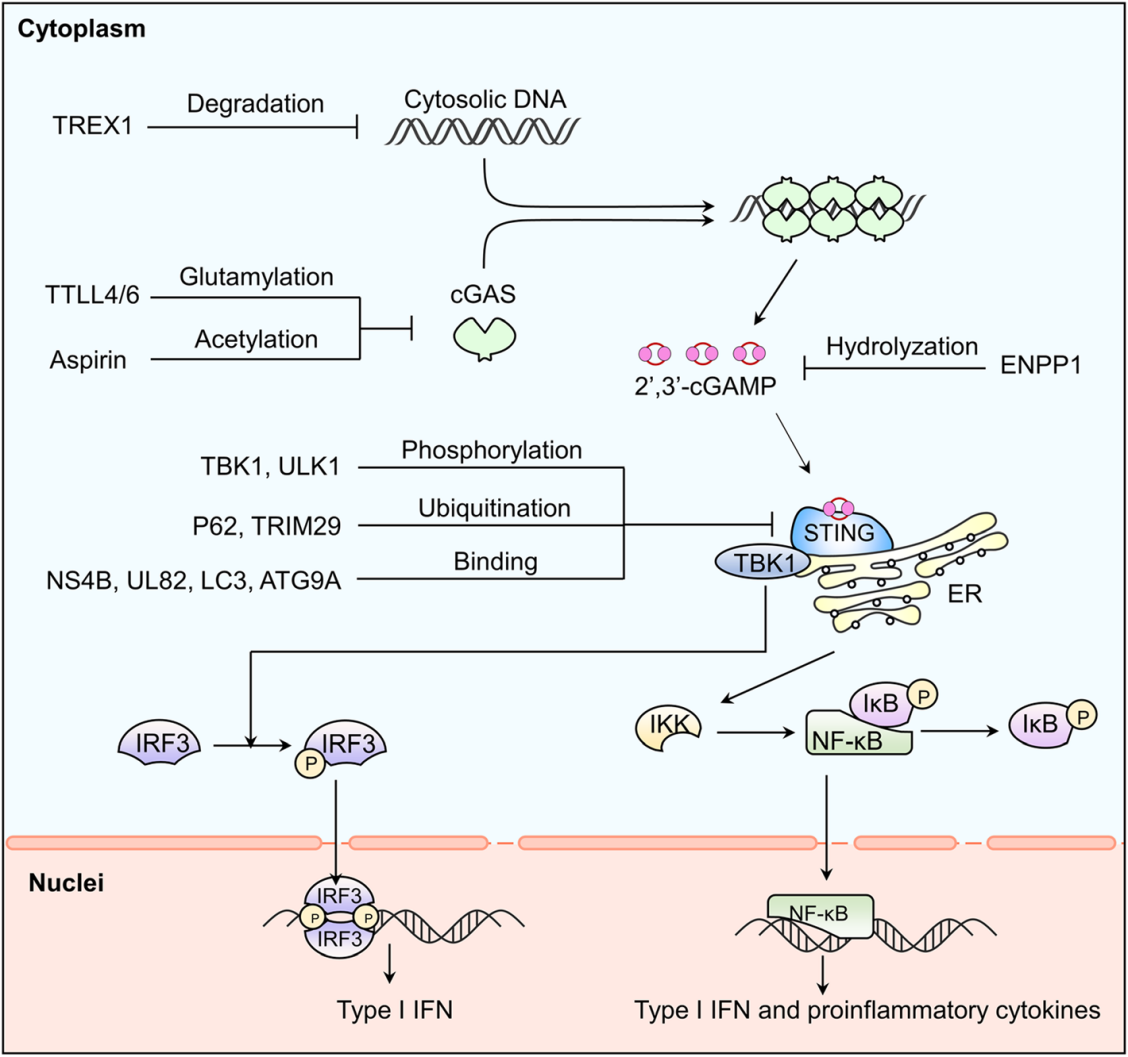
cGAS provoking pro-inflammatory reaction and its regulations. cGAS leads to a pro-inflammatory reaction through the classic cGAS-STING-IFN1 axis. And this axis can be regulated in different levels: dsDNA, cGAS, cGAMP, STING and downstream regulators

cGAS is a kind of cytosolic DNA sensor which initiates innate immune (Fig.1). Notably, cGAS and its downstream regulators form a major DNA-sensing mechanism, sensing foreign and self-DNA in the cytoplasm and sometimes in the micronucleus and nuclei as well [8]. The canonical cGAS/STING pathway starts with the activation of cGAS. cGAS is activated by accumulated cytosolic DNA and produces cGAMP as a second messenger. cGAMP then activates STING, an ER resident transmembrane protein [9]. Activated by cGAMP, STING will be transferred from ER to the Golgi body via the ER-Golgi intermediate compartment (ERGIC) and recruits TANK-binding kinase 1 (TBK1) [10]. Combined with STING, TBK1 will be delivered to lysosomal compartments to catalyze the phosphorylation of interferon regulatory factor 3 (IRF3) [11, 12]. Phosphorylated IRF3 will be dimerized and translocated into the nucleus to stimulate the expression of IFN-I and IFN-stimulated genes (ISGs). In parallel, STING also activates inhibitor of nuclear factor kappa-B (NF-κB) kinase (IKK). IKK phosphorylates and deactivates the inhibitor of NF-κB (IκB). NF-kB then is released from IκB and enters the nucleus, where it functions together with IRF3 and other transcription factors to induce the expression of interferons and inflammatory cytokines such as TNF, interleukin (IL)-1b and IL-6 [13]. STING may also directly bind to the cytosolic DNA but the pathological background is not fully elaborated [14]. Recent evidence has shown that the primordial function of STING is related to autophagy [15], as is proved in Zika virus infection of the Drosophila brain [16].

In addition to the classic cGAS/STING-IFN axis (Fig. 1), study has revealed that cGAS interacted with Beclin-1 to trigger autophagy, which would reduce cGAMP production independent of STING activation [17]. Similarly, activation of STING could be achieved in a cGAS-independent manner. Noncanonical STING signaling in response to etoposide-induced DNA damage could be activated by DNA-repair proteins ataxia telangiectasia mutated (ATM), poly-ADP ribose polymerase 1 (PARP1) and DNA binding protein interferon-γ-inducible factor 16 (IFI16) mediated NF-κB signaling in keratinocytes [18]. Similar regulatory processes can also be seen in some specific tumor models, such as in HCT116 colorectal carcinomas [19].

As a classic pathway, components of cGAS pathway, up and down stream regulatory factors and roles in autoimmune diseases and tumor immunity have been fully described and summarized [8, 20, 21]. In this review, we only briefly introduce these aspects as background information. Emphasis will be placed on the latest research on position-dependent cGAS function and non-immune physiological regulatory processes such as cellular senescence, programmed cell death (PCD), mitophagy Ca^2+^ homeostasis and ER stress and the different roles of cGAS/STING pathway in some human diseases.

## cGAS interacting with different sources of DNA

### The structure of cGAS

Like other proteins, cGAS has a C terminus (160–522) and a N-terminus (1 ~ 159). C terminus contains a nucleotidyltransferase domain and two DNA binding sites [22, 23] which assist cGAS dimerizes and binds to the sugar-phosphate backbone of double strand DNA (dsDNA) in the form of 2:2 [24, 25]. Single-stranded DNA (ssDNA) generated from reverse transcription weakly activates cGAS [8]. Specifically, unpaired guanosines are the necessary DNA structures for the activation of cGAS, suggesting that cGAS has the ability to recognize specific DNA sequence under certain circumstances. For example, cGAS can be activated effectively by the short (12- to 20-bp) human immunodeficiency virus type 1 (HIV-1) Y-form DNA in a sequence-dependent manner [26]. After binding to DNA, its nucleotidyltransferase domain transfers adenosine 5′-triphosphate (ATP) and guanosine 5′-triphosphate (GTP) to cGAMP and activates downstream pathways [27, 28].

For a long time, the functional significance of cGAS N-terminus (1 ~ 159) remains unclear [23]. However, researchers have recently taken a small step closer toward this. Du and Chen reported that N-terminus contributes to cGAS and DNA liquid droplets formation in physiological buffer and human cell lines, and therefore promotes the activation of cGAMP production [29]. N-terminus is also important for the subcellular localization of cGAS [30]. In the absence of DNA, N-terminus helps cGAS bind to the PI (4,5) P_2_ at the cell plasma membrane which reduces the sensitivity of cGAS to self-DNA. When the interaction between N-terminus and the plasma membrane disappears, cGAS will translocate into the cytoplasm and nucleus [30]. C-terminal domain 161 ~ 212 is crucial for cytoplasmic retention. While two nuclear localization sequences (NLS): N-terminal NLS1 (21 ~ 51) and C-terminal NLS2 (295 ~ 305) are needed for nuclear translocation [31].

### cGAS and cytosolic DNA

**Fig. 2:**
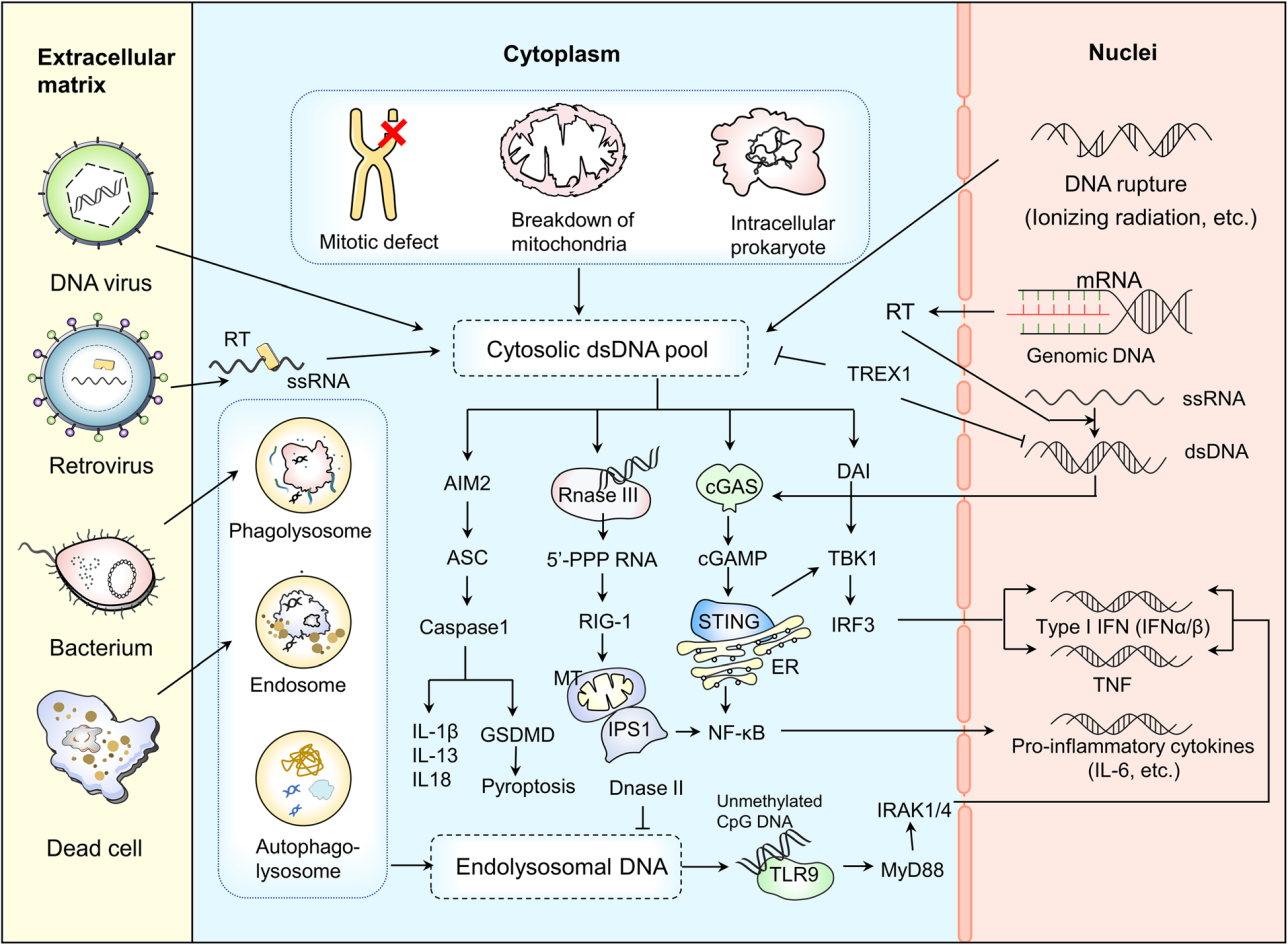
Source and function of cytosolic DNA. The cytosolic dsDNA pool is composed of DNA originating from retroviruses, DNA viruses, mitotic defects, mitochondria, intracellular prokaryotes and DNA rupture debris from the nuclei. In the cytosol, these DNA are either degraded by DNase III and TREX1 or sensed by DNA sensors. Once sensed by cytosolic sensors including AIM2, RNaseIII, cGAS and DAI, a cascade of reactions are triggered. Downstream signal molecules regulate the expression of immune-related genes, eventually leading to DNA clearance or cell death. In addition, DNA also comes from dead cells and bacteria. Membrane vesicles are formed by endocytosis and DNA is transported into the cell. DNase II is localized in lysosomes and digests DNA from pathogens and dead cells that end up in this cellular compartment. TLR9 directly binds with the remaining unmethylated CpG DNA and triggers downstream immune activation, inducing the expression of inflammation-related genes

The main sources of cytoplasmic DNA are as follows (Fig. 2) [32, 33]: (1) Intracellular pathogens infection, such as DNA viruses, retroviruses and intracellular prokaryotes; (2) Reactivation of endogenous retroviral sequences which codes a catalytically active retro transcriptase; (3) Imbalanced control of endogenous DNA such as mitochondrial breakdown, mitotic defects and DNA rupture; (4) Impaired ability to clear exogenous DNA; (5) Importing extracellular DNA-containing exosomes and/or micropinocytosis. Crystal structures of human cGAS and DNA-bound cGAS shows that cGAS is activated when two cGAS molecules and two dsDNA molecules compose together to form a ladder-like structure [34]. Bacterial and mitochondrial nucleoid proteins HU, mitochondrial transcription factor A (TFAM) and high-mobility group box 1 protein (HMGB1) support the recognition of dsDNA by elongating DNA sensing time via inducing the formation of U-turns and bends in DNA [35]. cGAS modified by other molecules also links tightly with the activity of dsDNA induced immune responses [36–39]. For example, polyglutamylases such as tubulin tyrosine ligase-like family member 6 (TTLL6) catalyzes the polyglutamylation of cGAS and hinders its binding with DNA, which can be canceled and reversed by the cytosolic carboxypeptidase 6 (CCP 6); Monoglutamylases such as TTLL4 catalyzes the monoglutamylation of cGAS and impedes the synthase of GAMP, which can be removed and recovered by CCP 5 [36]; Tripartite motif 56 (TRIM 56) E3 ubiquitin ligase monoubiquitinates cGAS at the Lysine 335 (K335), facilitating the binding of DNA and the synthesis of cGAMP by increasing the dimerization of cGAS [37]; The small ubiquitin-like modifier (SUMO) SUMOylates cGAS at K335, K372, and K382, restraining the following reactions, which can be reserved by sentrin/SUMO-specific protease 7 (SENP7) [38]; And also, Recently, both in vivo and in vitro studies have shown that GTPase-activating protein SH3 domain-binding protein 1 (G3BP1) binds to cGAS directly and helps cGAS bind with dsDNA by forming the large G3BP1-cGAS complexes [39].

Cytoplasmic DNA-induced cell death and immune response are self-defense against harmful substances from the internal and external environment. Drugs using DNA to induce immunity against tumors and infectious diseases are under developing and testing [40].

### cGAS and micronuclear DNA

**Fig. 3:**
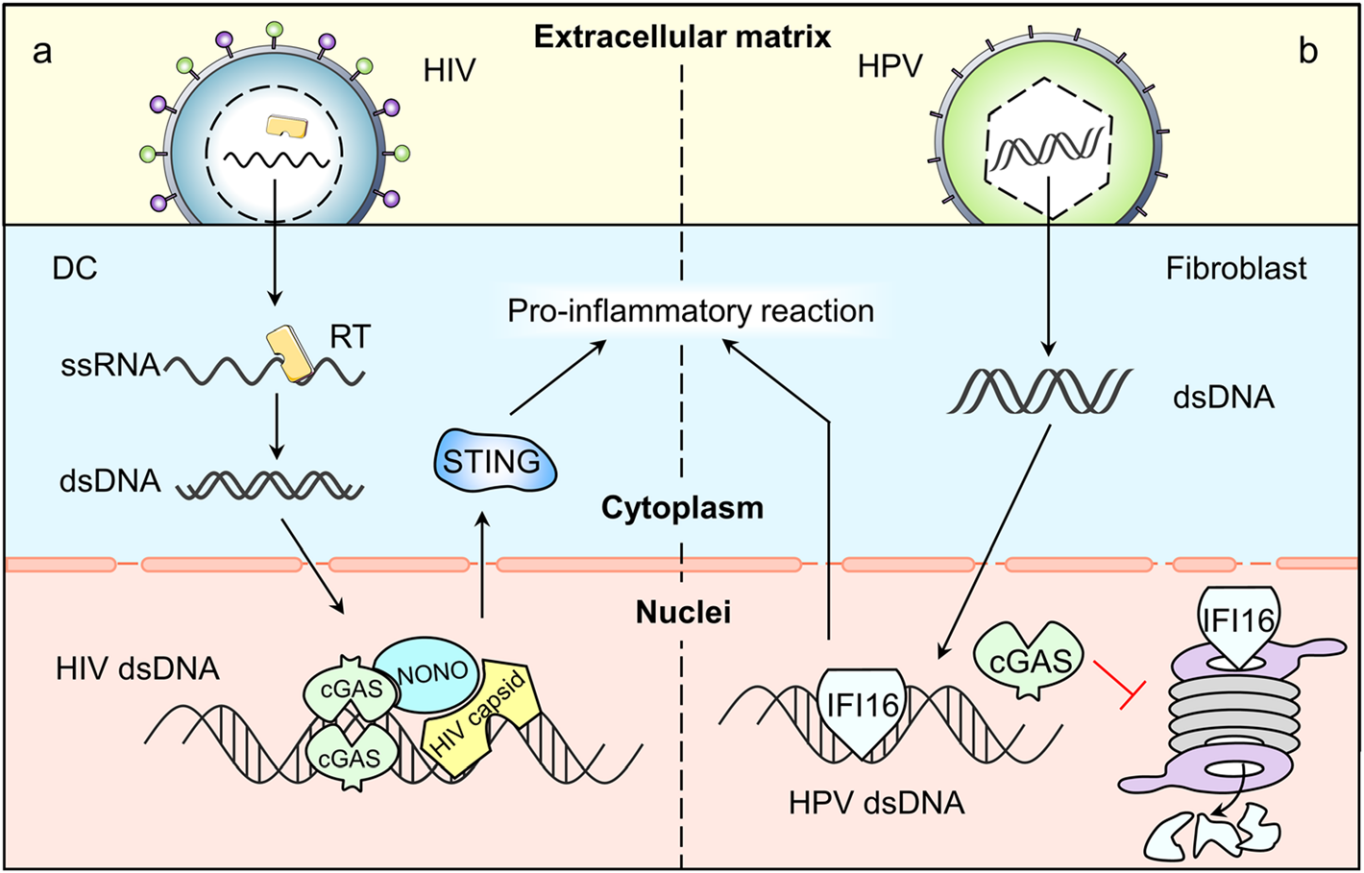
The generation of micronuclei and cGAS’s role in micronuclei.** a.** Micronuclei are generated when the genome is unstable during cell division, often associated with an abnormal nucleus in a daughter cell. **b.** After micronuclei form, the micronuclear envelope ruptures irreversibly. Then, cGAS enters into the micronuclei, binding to the chromatin and facilitating the downstream proinflammatory signals

Micronuclei is a cytoplasmic compartment which is composed of a membrane envelope and chromatin in it. Generally, micronuclei is regarded as an accurate indicator of genomic instability [41] (Fig. 3). It is formed when mitosis process encounters with mis-segregations of a whole or a part of a chromosome, accompanied with chromatin bridging and chromosomes/chromatin formation lagging behind [42, 43].

Some evidence shows that cGAS binds with chromosomes during mitosis [44]. But cGAS/STING pathway remains inactive, probably due to the tight-compacted structure of chromosomes [45]. It is noteworthy that cGAS will dissociate from chromosomes when mitosis is done. However, when micronuclei forms, a high level of cGAS shows up in the micronuclei during the interphase [46]. Then micronuclear cGAS mediates the downstream process [10, 22, 46] in a timely and cell-cycle dependent manner [47]. Under such circumstances, micronuclei acts as a reservoir of immunostimulatory DNA, which may function as a compensative cell cycle checkpoint [48, 49].

Activation of cGAS by micronuclear DNA requires the entry of cGAS into micronuclei. Micronuclei forms when lagging chromosome separates from the primary nucleus and has its own membrane [41]. Then, the micronuclear envelope shatters irreversibly which occurs at a random phase. That means it is not confined to a specific phase but sensitive to DNA damage [44]. This failure of membrane integrity is associated with the reduction of lamin B1 [41, 47], which may reverse by nuclear β-dystroglycan (β-DG) [50]. As a consequence, the cytosolic cGAS gains the access to micronuclei and thereby binds to chromatin and initiates downstream proinflammatory responses [47].

As we discussed above, the formation of micronuclei is an important mark of genomic instability. And genomic instability caused by the activation of multipolar mitotic spindles [50], the production of DNA-damage agents [51], centrosome abnormalities [52], telomeres dysfunctions [53–55] andp53/p21 mutation [56] are hallmarks of cancer and cellular senescence. The disruption of the micronuclei caused by aberrant accumulation of the endosomal sorting complex required for transport-III (ESCRT-III) complex or the mutant prelamin A (progerin) accentuates DNA damage and enhances pro-inflammatory responses via cGAS/STING pathway [57–59]. The formation of micronuclei has a dual function. On the one hand, abnormal accumulation of micronuclei is related to cancer and aging. On the other hand, the micronuclear dsDNA activates cGAS-mediated immune response, which is an important innate immune surveillance mechanism for the clearance of cancer cells and senescent cells.

### cGAS and nuclear DNA

#### cGAS interacting with endogenous DNA in nuclei

**Fig. 4:**
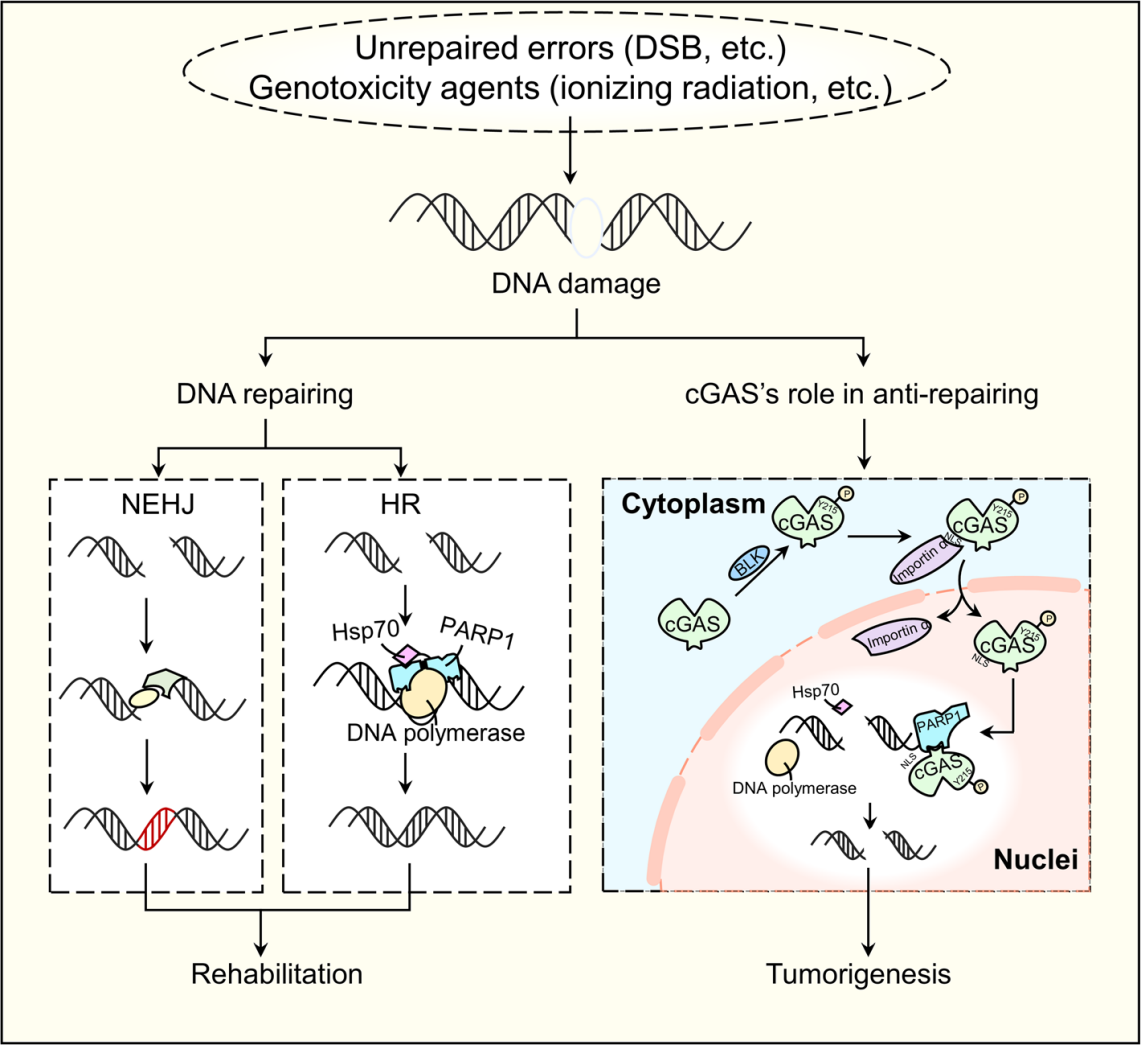
cGAS interacting with endogenous DNA in nuclei. Naturally, DNA damage caused by unrepaired errors or genotoxic agents can be corrected by two basic DNA DSB repair mechanisms: NHEJ and HR. However, in some circumstances, when DNA is damaged, cGAS can be phosphorylated by BLK and then translocated into the nucleus with the help of importin-α. In nucleus, the phosphorylated cGAS interacts indirectly with PARP1, impeding HR and thus promoting tumorigenesis

Micronuclear cGAS works as a supervisor. When unstable genetic material appears, cGAS will initiate its downstream pro-inflammatory responses, linking the genome instability with innate immune. However, in some cases, cGAS enters into nuclei and inhibits DNA repair when DNA damage happens, and therefore playing a tumorigenic role adversely [31] (Fig. 4).

DNA damage arises more frequently when exposed to genotoxic therapies such as chemotherapies [60]. Among all forms of damage, the double-stranded breaks (DSBs) of DNA strains are the severest type [61]. These damages can be rescued by two basic DNA DSB repair ways: homologous recombination (HR) and nonhomologous end joining (NHEJ) [62–64]. However, studies have confirmed that this repair process can be interrupted by cGAS via binding with chromosomes and initiates immune response [31]. The underlying mechanism and significance of cGAS binding with chromosomes have not been fully elucidated. One thing we can confirm is that it is the NLS-mediated entry of cGAS rather than the low-level physiological accumulation of cGAS that activates innate immune [65]. More in-depth research is needed to better understand this process.

#### cGAS interacting with exogenous DNA in nuclei

Viruses live a highly parasitic live. They use host’s resources and organelles as production materials and workshops and own genetic material as templates to proliferate. In response, mammals have a set of complicated mechanisms to detect and kill those viruses. At the same time, viruses never stop attempting to elude and defect the surveillance system of the host.

**Fig. 5:**
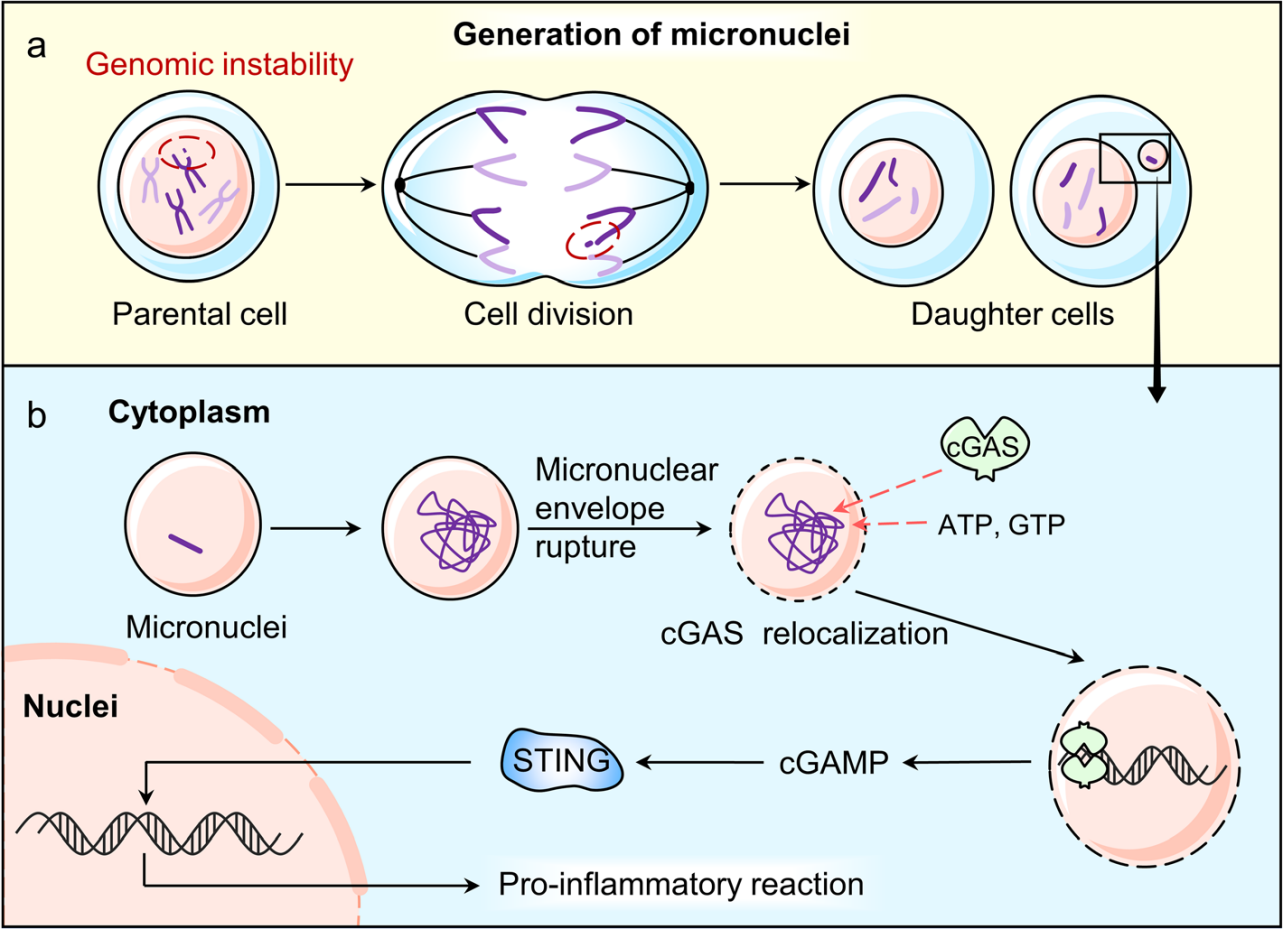
cGAS interacting with exogenous DNA in nuclei. a. HIV’s ssRNA enters into the DC and is synthesized into HIV dsDNA in the cytoplasm. Then, the dsDNA is admitted into the nuclei, where capsid protein binds directly to NONO, promoting cGAS’s recognition of HIV dsDNA. b. HPV’s dsDNA is transmitted into the fibroblast and then enters into the nuclei. The nuclear cGAS prolongs the half-life of IFI16 (another DNA sensor) by promoting the degradation of proteasomes

The innate immune system of human body reacts immediately in response to virus infections. Pattern recognition receptors (PRRs) recognize the conservative pathogen-associated molecular patterns (PAMPs) or host damage associated molecular patterns (DAMPs) [66], followed by cascade signaling reactions. In this process, cGAS plays a pivotal role in detecting viruses and activating dendritic cells (DCs) and macrophages [67, 68]. The recognition process often starts in the cytoplasm, sometimes in nucleus (Fig. 5).

HIV infection is a typical example of nuclear recognition process. Earlier studies show that cGAS is vital for the recognition of retro-transcriptional synthetic HIV dsDNA by immune cells both inside and outside the cell nuclei [26]. To active cGAS, Non-POU domain-containing octamer-binding protein (NONO) first binds to capsid protein DCs nucleus. Then, NONO interacts with cGAS to form a complex which in turn promotes the recognition of HIV-2 DNA and the initiation of nuclear cGAS induced STING activation [69].

cGAS also participates in the recognition of nuclear DNA through an indirect, STING independent way. This is achieved by helping maintain the stability of other DNA sensors such as IFI16 [70]. When infected by human papillomavirus (HPV), normal human fibroblasts will develop a certain mechanism to facilitate IFI16 in binding directly with HPV DNA in the nucleus [70]. At the same time, the existence of cGAS prolongs the half-life of IFI16, may by promoting the degradation of proteasome [70]. Though more research is needed to confirm the role of cGAS in this process, we may able to develop cGAS as an immune enhancer to support our body in defending virus.

## Regulation of cGAS/STING pathway

Regulation of cGAS/STING pathway has been thoroughly discussed elsewhere [8]. Here we only give a brief summary and add some new findings (Fig. 1). The regulation is complex and multidimensional, mainly from the following aspects:

1. Degradation of cytosolic dsDNA. To avoid the cellular disorder triggered by cytosolic DNA, TREX1 degrade mis-localized DNA and maintain the balance of homeostasis and inflammation response [4].
2. Regulation of cGAS. Transcriptional, epigenetic regulations and post-translational modifications are all involved. Besides these aspects discussed in the referent paper, chemical modifications also participate in regulating the activity of cGAS. For instance, Aspirin robustly inhibits cGAS activation and cGAS-mediated IFN production by directly acetylating the cGAS at K384 and/or K394 and K414 [4].
3. Regulation of cGAMP. Activity and location of cGAMP are controlled by ecto-nucleotide pyrophosphatase/ phosphodiesterase 1 (ENPP1) and intercellular transmission respectively [71]. In vivo study is still limited but the in vitro study shows that overexpression of ENPP1 significantly lowers cGAMP level and reduces production of IFN-β and NF-κB in porcine cells infected with pseudorabies virus (PRV) [72].
4. Modification of STING. Post-translational modifications, trafficking degradation and binding affinities with cGAMP are involved. Phosphorylation by serine/threonine UNC-51-like kinase (ULK1) and TBK1 and ubiquitination by ubiquitin-binding protein p62 lead to the degradation of STING [73, 74]. Interestingly, the function of p62 is dependent on TBK1 and IRF3, which indicates negative feedback on the attenuation of signaling [73]. Moreover, the tyrosine-protein phosphatase nonreceptor type (PTPN) 1 and 2 dephosphorylate STING at Y245 which promotes its 20S proteasomal degradation [75]. Additionally, DNA virus infection triggered the ubiquitination of STING by upregulating TRIM29 [76].

In addition to modification, regulators inhibit STING by directly binding to STING. Such as autophagy proteins, including microtubule-associated protein 1 light chain 3 (LC3) and autophagy-related protein 9a (ATG9A) [77]. Pathogenic proteins, such as Hepatitis C virus non-structural 4B (NS4B) protein and human cytomegalovirus (HCMV) tegument protein UL82, directly interact with STING to reduce STING activity [78, 79]. Meanwhile, DNA tumor viruses, such as HPV18 and human adenoviruses 5 (hAd5), inhibit the activation of cGAS/STING pathway by producing oncoprotein binding STING [68]. Moreover, the Ca^2+^ sensor stromal interaction molecule 1 (STIM1) binds to STING and elongates STING retention with ER, consequently hindering the following cascade [80].

## Roles of the cGAS/STING pathway in physiological regulatory processes

**Fig. 6:**
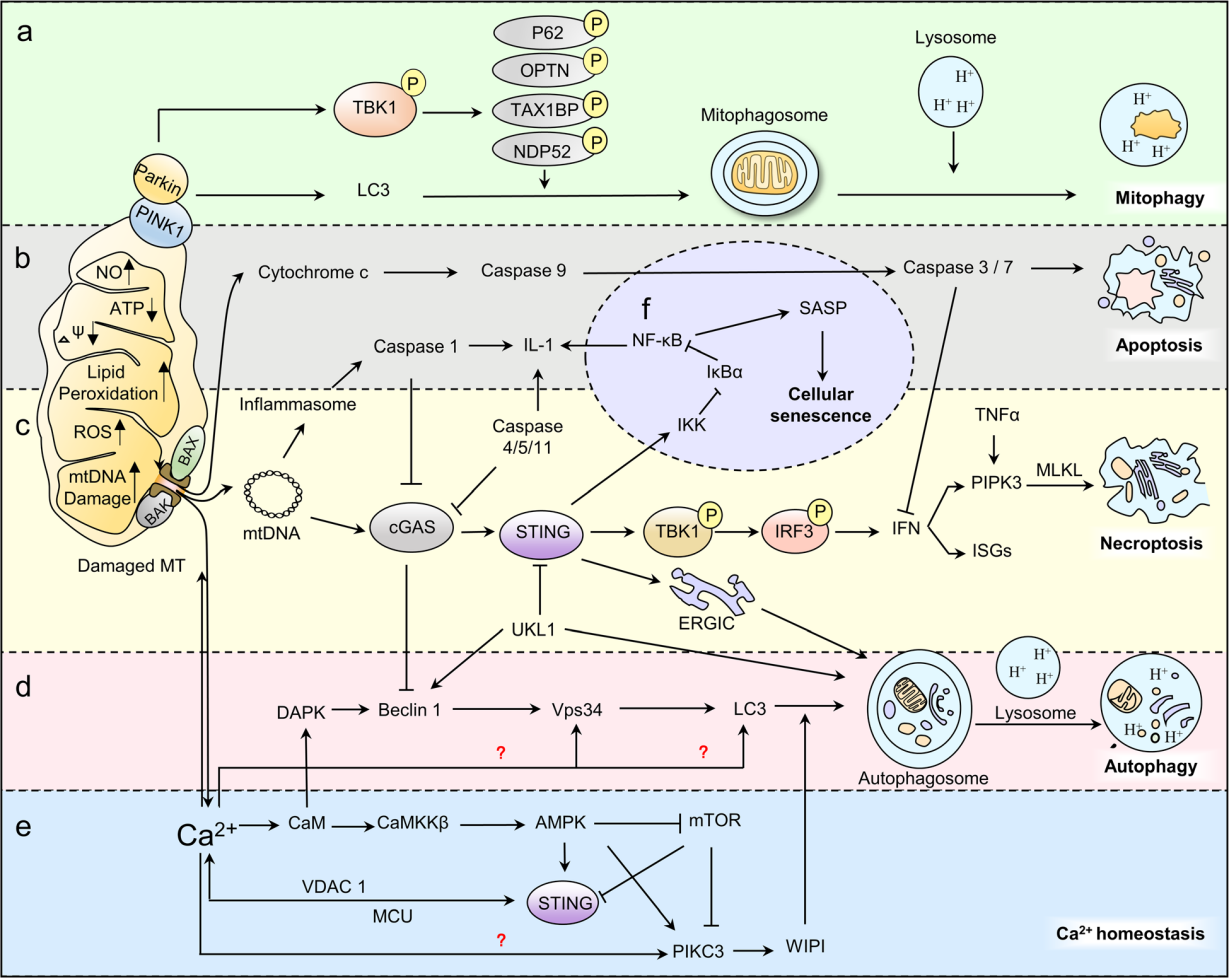
Regulatory role of cGAS–STING pathway in physiological processes. cGAS/STING participates in regulating mitophagy (**a**), apoptosis (**b**), necroptosis (**c**), autophagy (**d**), cytoplasmic Ca^2+^ homeostasis (**e**), production of SASP and cellular senescence (**f**)

A bulk of studies have exhaustively summarized the role of cGAS/STING pathway in regulating antipathogenic and antitumor responses and the adaptive changes of cancerous cells and pathogens to escape cGAS supervision [8, 81–84]. As we explore and know more about this pathway, we are able to find more in other aspects. Therefore, here we focus on other newly found and also important aspects, including its role in nuclei, senescence, mitochondrial dysfunction, ER stress and Ca^2+^ homeostasis (Fig. 6).

### Cellular senescence

Cellular senescence is first proposed by Hayflick and Moorhead in 1961, which is defined as irreversible cell-cycle arrest that occurs when cells experience potentially oncogenic stress [85]. According to different incentives, such as telomere shortening, certain oncogenes and chemotherapeutic drugs or ionizing radiation, senescence can be subdivided into replicative senescence, oncogene-induced senescence (OIS) and therapy-induced senescence (TIS) respectively [86]. The p53/p21 and p16^INK4a^/pRB pathways are responsible for senescence related growth arrest [87]. Senescent cells, though fail to initiate DNA replication, remain metabolically active and secret sorts of proteins, including proteases, various growth factors, cytokines and chemokines with proinflammatory properties. Collectively, these secretions are termed as SASP that have complex effects on cell behaviors, especially in aging and tumorigenesis [87, 88]. As we mentioned before, mtDNA and micronuclei are two sources of cytoplasmic dsDNAs. Considering that mitochondrial dysfunction and genomic instability are typical features of aging [89], it is not difficult to link cGAS/STING with cellular senescence.

In senescent cells, several factors contribute to the accumulation of cytoplasmic DNA and the activation of cGAS: (1) Loss of the nuclear lamina protein Lamin B [90]. Decreased Lamin B is a hallmark of senescence which leads to collapse of the nuclear envelope, triggering release of chromatin fragments from the nucleus to the cytosol, termed as cytoplasmic chromatin fragments (CCF) [91]. (2) Leakage of mtDNA. Accumulated oxidative damage to mitochondrial membrane proteins and lipids leads to increased membrane permeability. Membrane break up results in the leakage of mtDNA to cytoplasm [92]. (3) Downregulation of TREX1 [93]. TREX1 is responsible for degrading the double-stranded and single-stranded DNA in the cytoplasm to prevent accumulation of DNA. (4) Upregulated long-interspersed element-1 (LINE-1, also known as L1) [94]. LINE-1 is a retro-transposable element which transcribes mRNA to cDNA and causes cytoplasmatic DNA accumulation. (5) Increased MUS81protein [95]. MUS81 is a structure-specific endonuclease that resolves inter-strand DNA structures such as stalled replication forks and Holliday junctions. MUS81is engaged in changing nuclear DNA into cytoplasmic forms which causes elevated cytoplasmic DNA [96].

Binding with accumulated self-derived DNA fragments in senescent cells, cGAS then activates downstream expression of NF-κB and triggers SASP production in senescent cells [86]. Studies find that suppressing either cGAS, STING or NF-κB in mouse and human cells abrogates the expression of senescence-associated inflammatory genes in response to DNA damaging agents such as etoposide and ionizing irradiation [44, 97]. The secretion of several SASP factors is regulated by cGAS, including IL-6, a critical controller of autocrine senescence, CXCL10, a cGAS-dependent IFN-stimulated gene, TNF-α and several chemokines [98, 99]. The connection between cGAS/STING pathway and SASP regulation remains largely unknown. However, the consistent performance in in between reflects the existence of close relationship. Future work could focus on the molecular mechanism of cGAS/STING pathway in senescence and aging-related diseases to see if GAS also has a regulatory effect such as neurodegenerative diseases, osteoarthritis, cardiovascular diseases, etc. (Fig.6f).

### PCD

Depending on different endogenous and exogenous threats, cells have 3 different fates: (1) Restore and back to normal function if the threats are successfully eliminated; (2) Enter senescence if the damage is persistent but tolerable; (3) Undergo PCD or necrosis if the damage is beyond management. The death of infected cells is an important defense that limits viruses to subvert the cellular machinery for their own replication. This part has been well established [100, 101]. Here we are going to talk about latest understanding of cGAS/STING in regulated cell death (RCD).

Based on the macroscopic morphological alterations and where dead cells and their fragments are disposed, RCD is detailed classified into different subtypes [102, 103], apoptosis, autophagy and necroptosis. Intrinsic apoptosis is induced by cellular stress and starts with the activation of apoptotic caspases (caspase-3, − 6, − 7, − 8, and − 9) [104]. Exposed to stress, mitochondrial outer membrane permeabilization (MOMP) is formed by Bax (Bcl-2-associated x protein) / Bak (Bcl-2 antagonist killer 1) channel. Mitochondrial contents such as cytochrome c is then released form MOMP into the cytosol where it binds to the NLR protein apoptotic protease activating factor 1 (APAF1). This binding forms the apoptosome — an activating platform for the initiator caspase 9 [105, 106]. Activated caspase 9 in turn activates the effector caspases, caspase 3 and caspase 7 [107]. Executioner caspase 3 and 7 trigger a cascade of proteolytic events that culminate in the demise of the cell through apoptosis. cGAS participates in apoptosis via regulating caspase-3. Activated by apoptosis signals, caspase-3 cleaves and inactivates cGAS, mitochondrial antiviral-signaling protein (MAVS), and IRF3 to suppress cytokine and type I IFN production in order to keep immunologically silent [108–110]. While caspase inhibition prompts the widen of BAX/BAK-mediated pores which leads to the extrusion of unstructured mitochondrial inner membrane [111]. Mitochondrial inner membrane permeabilization facilitates mtDNA release into the cytoplasm and activate cGAS/STING signaling and IFN synthesis, enabling cell death-associated inflammation [112]. The inflammatory caspases-1, − 4, − 5 and − 12 also influence cGAS function. Under DNA virus infection, caspase-1 interacts with cGAS, cleaving it and dampening cGAS/STING-mediated IFN production. Caspase-4, 5, and 11 cut cGAS under non-canonical inflammasome activation [113, 114]. Understanding the complex regulatory network between cGAS and caspases at the intersection of programmed cell death and innate immune regulation is helpful for better understanding related human diseases [100, 115] (Fig. 6b).

Autophagy is a self-degradative process that allows the recycling of cellular components. It is also a final barrier against oncogenic transformation that restricts chromosomal instability during replicative crisis [116]. Basic autophagy process have been summarized and generally accepted [117]. Here we introduce the newly found relationship between autophagy and cGAS. In this process, interferon induction is not indispensable. For example, in macrophages, activated STING and the kinase TBK1 lead to ubiquitin-mediated selective autophagy pathway, limiting *M. tuberculosis* growth during infection independent of IFN production [118, 119]. In addition, cGAS can directly bind with Beclin-1 autophagy protein and release the negative autophagy regulator Rubicon from the Beclin-1 complex. This interaction activates downstream phosphatidylinositol 3-kinase class III and induces autophagy to remove cytosolic pathogen DNA independent of TBK1 activation [17]. Another TBK1 independent way relies on the formation of ERGIC. After binding with cGAS, STING buds from the endoplasmic reticulum into coat protein II (COP-II) vesicles then forms the ERGIC. The ERGIC serves as the membrane source for WD-repeat PtdIns (3) P effector protein 2 (WIPI2) recruitment and LC3 lipidation. Autophagosomes that target cytosolic DNA or DNA viruses then formed and merge with the lysosome. Besides, the discovery in sea anemone prompts us that autophagy induction is an ancient and highly conserved function of the cGAS/STING pathway that even pre-dates the emergence of the type-I interferon pathway in vertebrates [15]. Autophagy induced via these pathways prevents replication of pathogens by eliminating the infected cells, protecting the body against pathogen attack. Other roles of cGAS/STING induced autophagy include protecting liver from ischemia-reperfusion injury [120] and restricting chromosomal instability during replicative crisis. This replicative check point also serves as a final barrier against oncogenic transformation by eliminating precancerous cells with disrupted cell cycle checkpoints [121] (Fig. 6d).

Necroptosis is a lytic form of PCD that involves the swelling and rupture of dying cells. cGAS/STING is able to induce necroptosis in bone marrow derived macrophages via type I IFN signaling pathways, which synergizes to trigger RIPK3 (receptor interacting protein kinases 3) and MLKL (Mixed lineage kinase domain-like) driven necroptosis independent of caspase-8 function [122, 123]. Moreover, cGAS/STING activated by mitochondrial DNA has been suggested to amplify necroptosis via a TNF-dependent mechanism [124, 125] (Fig. 6c).

Substantial crosstalk exists between different cell death pathways ensuring that these signaling pathways are well regulated. More studies are required to further explore the interconnection among these pathways and how cGAS/STING signaling toggles in transcriptional responses, different forms of RCD, anti-neoplastic transformation and anti-infection reactions.

### Others

Mitophagy is a selective form of autophagy controlled by the Pink1-Parkin pathway or the mitophagic receptors Nix and Bnip3. The physiological role of mitophagy is specifically removing damaged or excessive mitochondria [126]. cGAS/STING does not participate in regulating mitophagy directly. But when damaged mitochondria failed to be removed, stress from mitochondrial DNA mutations activates the proinflammatory cGAS/STING pathway which may contribute to several age-related neurodegenerative diseases, for example, Parkinson’s and Alzheimer’s disease [127, 128]. The engagement of cGAS and subsequent mtDNA-induced STING-mediated type I IFN production can be suppressed by apoptotic caspase 9 and downstream caspase 3 and 7, rendering mitochondrial apoptosis immunologically silent [108, 110] (Fig. 6a).

Though many unknowns remain in the regulation among cGAS, Ca^2+^ homeostasis and ER stress, the existing research findings show promising results for future investigation. Here we list the brief summary of these findings: (1) STING influences the intracellular Ca^2+^ level via affecting the mobilization of ER Ca^2+^ pool. As we put above, STING is found on the contact sites between the ER and mitochondria where Ca^2+^ is exchanged between these two organelles via channels like voltage-dependent anion channel 1 (VDAC1) and mitochondrial Ca^2+^ uniporter (MCU). STING deficiency augments the translocation of stromal interaction molecule 1 (STIM1), a Ca^2+^ sensor. Then the depletion of ER Ca^2+^ stores trigger Ca^2+^ entry [80]. (2) Intracellular calcium is a rheostat for the STING signaling pathway. Reductions in cytosolic Ca^2+^ and the mitochondrial export of Ca^2+^ reduced the activation of NF-κB and IRF3. While increased intracellular Ca^2+^ from ER and mitochondria promotes STING activation via two independent Ca^2+^-calmodulin dependent pathways: AMPK (Adenosine 5′-monophosphate (AMP)-activated protein kinase) and CAMKII (Ca2+/calmodulin-dependent protein kinase II) pathways [129]. (3) ER stress activates STING pathway. ER stress, either induced by alcohol or co-stimulation with thapsigargin (the sarcoplasmic endoplasmic reticulum calcium ATPase (SERCA) pump inhibitor), enhances STING signaling and augments IFN production [130, 131]. Besides, STING activates ER stress and the unfolded protein response (UPR) through a novel motif termed as “the UPR motif”, which is located in the helix aa322–343. Long-lasting STING-mediated ER stress and disruption of calcium homeostasis primes T cell death by apoptosis [132–134] (Fig.6e).

## Roles of the cGAS/STING pathway in human diseases

Generally, cGAS is allocated to a limited subcellular area that is free of self-DNA. Several endogenous nucleases participate in maintaining self-DNA level under the threshold of receptor activation. However, under pathologic circumstance, self-DNA is exposed to cGAS. This will lead to abnormal activity of the cGAS/STING pathway, causing autoinflammation and autoimmune disease and even inflammation-associated cancers. Abnormal activation of cGAS/STING pathway in inflammatory and autoimmune diseases is well discussed in other papers [8, 21, 135]. Here we take Aicardi-Gourtières syndrome (AGS), a typical disease caused by the excessive activation of cGAS, as an example to illustrate the mechanism. AGS is caused by causal mutations in any one of several key genes, including TREX1, RNASEH2A (Ribonuclease H2 subunit A), RNASEH2B, RNASEH2C (which together encode the Ribonuclease H2 enzyme complex), SAMHD1 (Sterile alpha motif and histidine-aspartic acid domain-containing protein 1), ADAR1 (adenosine deaminase acting on RNA 1) and IFIH1 (interferon induced with helicase C domain 1, also known as MDA5) [136–140]. These genes are responsible for cleaning ectopic DNA, lack of which causes inappropriate accumulation of self-derived nucleic acids, sustained activation of cGAS and excessive production of type I interferons.

Apart from self-DNA-driven inflammation, other cGAS/STING pathway induced responses also participates in human diseases [44, 47, 141–144]. For example, mutations in gene ATG16L1 promote the production of IL-22 in the intestinal epithelium through cGAS/STING pathway, which result in excessive epithelial cell death and inflammatory bowel disease (IBD) [145]. The connection between the cGAS pathway and aging provides a new topic for us. Existing study has identified the cGAS/STING pathway as a sensor of senescence-associated DNA damage and trigger of inflammation in early age-related macular degeneration [146]. More research is needed on the relationship between cGAS/STING pathway and senescence-associated human diseases such as neurodegenerative diseases, degenerative arthritis and cardiovascular diseases.

Defective cGAS/STING signaling is closely associated with oncogenesis, immune evasion and tumor metastasis [147, 148]. Antineoplastic role of cGAS has been found in multiple mouse tumor models, including colon, brain, skin, pancreatic, liver, breast, and B cell malignancies [149, 150]. These protective effects are achieved mostly through IFN-induced immune responses and in few cases via autophagy [121, 151]. cGAS/STING pathway is also related to tumor microenvironment remodeling [82, 152] and the production of anti-tumor cytokines such as indoleamine 2,3-dioxygenase (IDO), IL-10 and ISGs, together inhibiting tumor growth and improving the survival [150, 153]. In vitro human study shows that targeting DNA damage response promotes antitumor immunity through STING-mediated T-cell activation in small cell lung cancer [154]. In vivo human-related research has not been performed, but we can assume the connection based on exiting in vitro studies.

Based on the anti-tumor role of cGAS/STING pathway, people began to design STING agonists and cyclic dinucleotide derivatives for tumor treatment. A novel synthetic cyclic dinucleotide, ADU-S100, has been promoted to phase Ib clinical trials in patients with diverse, solid, accessible tumors for achievable intra-tumoral delivery. Antitumor effects of ADU-S100 have been observed in PD-1–naive TNBC and PD-1–relapsed/refractory melanoma [155, 156]. Synthetic derivatives demonstrate a strong ability of inducing IFN-β in both murine BMDMs and primary human cells and forming antitumor immunological memory following tumor regression [153]. Contrary to the results mentioned above, activation of the cGAS/STING pathway is found in tolerogenic responses. By inducing indolamine 2,3-dioxygenase, cGAS/STING pathway promotes the growth of tumors with low antigenicity [157]. And a pan-cancer human study which analysis the association between the expression of cGAS/STING and immune cell infiltration shows that the upregulated cGAS/STING signaling is negatively correlated with the infiltration of immune cells in some tumor types [158]. Therefore, it is necessary to fully evaluate the function of cGAS/STING signaling in cancer immunity before the application of the STING agonist-based anticancer immune therapy [158].

cGAS/STING surveillance is a main part of antiviral responses, achieved mainly through the production of IFN. In addition to an initial virus-induced inflammatory cascade, cGAS/STING effectively engages a potent localized immune response via cGAMP transfer [159]. Autophagy is another effective anti-viral process that maintains cellular homeostasis by orchestrating immunity upon viral infection [160]. For example, ZIKV infects mature neurons in fly brain which induces Rel/NFKB inflammatory signaling. Rel/NFKB activates the expression of STING which then activates antiviral autophagy to restrict ZIKV infection [16, 161]. Recent study even reveals that autophagy induction in response to stimulation by cGAMP is a primordial function of the cGAS/STING pathway that pre-dates the emergence of the type-I interferon pathway in vertebrates [15]. More studies are needed to understand the role of cGAS/STING in anti-viral infection via induction of autophagy.

## Conclusions and perspectives

cGAS/STING is a well-studied signaling pathway that participates in sensing abnormal subcellular localization of DNA and mediating protective immune defense against infection. Over the past few years, studies have established the basic framework and mechanisms of this DNA-sensing pathway. However, the investigation of cGAS pathway in immunomodulation and antitumor therapy has attracted a lot of attention and its critical roles in other areas are overlooked. Accumulating evidence indicates that the physiological and pathological regulatory effects of cGAS/STING pathway extends far beyond “traditional” antimicrobial immunity. In this review, we summarize the current finds of cGAS/STING pathway in a broad repertoire of cellular processes, including mitochondrial function, ER stress, Ca^2+^ homeostasis, cellular senescence, PCD, and metastasis. Based on its broad regulatory roles, we could see the therapeutic application of cGAS/STING pathway in some age-related diseases such as neurodegenerative diseases, osteoarthritis, cardiovascular disease, chronic kidney and pulmonary disease. These diseases, like tumors, have abnormal elimination of pathological tissue or the alienation of normal tissues. Developing specific inhibitors of the cGAS/STING pathway to identify its role in these human diseases will be an important and exciting task in the future.

Undeniably, several questions remain to be answered. First is about aging and cellular senescence which are believed to participate in the pathology of many diseases. Accumulated DNA damage, genomic instability, mtDNA release are ubiquitous in senescent cells. As we talked above, cGAS pathway is highly likely to sense these DNA segments and regulate SASP production. However, it is unlikely to be achieved through the classic pathway as increased IFNs are not normally seen in natural aging process. Other downstream regulators are waiting to be found to better explain this regulatory role. Ca^2+^ homeostasis is another interesting part because ER stress, mitochondrial dysfunction and PCD are all related to cellular Ca^2+^ level. STING is located on ER membrane near the ion exchange channels between ER and mitochondria. Whether and how STING affects the on and off of these channels and the consequence of this regulation are largely unknown. Faced with internal and external pressure, how cells determine the balance between caspase-induced apoptosis and cGAS-induced IFN production is still un clear. Besides, how STING induces autophagy under virus infection is unknown. In addition, the regulation of this pathway, signaling mechanism at each step and the possible crosstalk with other pathways require more studies to identify.

In sum, we thoroughly summarize the broad roles of cGAS/STING pathway in several critical cellular processes. Maintaining the delicate balance between aging, immunity and proliferation is a necessary guarantee for cell living and functioning. Future research on cGAS could focus more on the role of cGAS in balancing these three aspects, interactions with other related regulating pathways and applications in human disease treatments.

## Data Availability

Not applicable.

## Abbreviations

ADAR1: Adenosine deaminase acting on RNA 1
AGS: Aicardi-Gourtières syndrome
AIM2: Absent in melanoma 2
AMPK: Adenosine 5′-monophosphate (AMP)-activated protein kinase
APAF1: Apoptotic protease activating factor 1
ASFV: African swine fever virus
ATG9A: Autophagy-related protein 9a
ATM: Ataxia telangiectasia mutated
ATP: Adenosine 5′-triphosphate
Bak: Bcl-2 antagonist killer 1
Bax: Bcl-2-associated x protein
BLK: B-lymphoid tyrosine kinase
CAMKII: Ca2+/calmodulin-dependent protein kinase II
CCF: Cytoplasmic chromatin fragments
CCP 6: Cytosolic carboxypeptidase 6
cGAMP: Cyclic GMP-AMP
cGAS: Cyclic GMP-AMP (cGAMP) synthase
COP-II: Coat protein II
DAMPs: Damage associated molecular patterns
DCs: Dendritic cells
DNases: Deoxyribonucleases
DSBs: Double-stranded breaks
dsDNA: Double strand DNA
E: Glutamic acid
ENPP1: Ecto-nucleotide pyrophosphatase/ phosphodiesterase 1
ER: Endoplasmic reticulum
ERGIC: Endoplasmic reticulum–Golgi intermediate compartment
ESCRT-III: endosomal sorting complex required for transport-III
G3BP1: GTPase-activating protein SH3 domain-binding protein 1
GTP: Guanosine 5′-triphosphate
hAd5: Human adenoviruses 5
HCMV: Human cytomegalovirus
HIV-1: Human immunodeficiency virus type 1
HMGB1: High-mobility group box 1 protei
HPV: Human papillomavirus
HR: Homologous recombination
HSV-1: Herpes simplex virus 1
IBD: Inflammatory bowel disease
IDO: Indoleamine 2,3-dioxygenase
IFI16: Interferon-γ-inducible factor 16
IFIH1: Interferon induced with helicase C domain 1
IFN-I: Type I interferon
IKK: Inhibitor of nuclear factor kappa-B kinase
IL: Interleukin
IRF3: Interferon regulatory factor 3
ISGs: IFN-stimulated genes
IκB: Inhibitor of NF-κB
K: Lysine
LC3: Light chain 3
LINE-1: Long-interspersed element-1
MAVS: Mitochondrial antiviral-signaling protein
MCU: Mitochondrial Ca2+ uniporter
MDA5: Melanoma differentiation-associated 5
MLKL: Mixed lineage kinase domain-like
MOMP: Mitochondrial outer membrane permeabilization
mtDNA: Mitochondrial DNA
NF-κB: Nuclear factor kappa-B
NHEJ: Nonhomologous end joining
NLRs: NOD-like receptors
NLS: Nuclear localization sequences
NONO: Non-POU domain-containing octamer-binding protein
NS4B: Non-structural 4B
OIS: Oncogene-induced senescence
PAMPs: Pathogen-associated molecular patterns
PAR: Poly-ADP ribose
PARP1: Poly-ADP ribose polymerase 1
PCD: Programmed cell death
PRRs: Pattern recognition receptors
PRV: Pseudorabies virus
PTPN: Tyrosine-protein phosphatase nonreceptor
RCD: Regulated cell death
RIG-I: Retinoic acid-inducible gene I
RIPK3: Receptor interacting protein kinases 3
RLRs: RIG-I-like receptors
RNASEH2A: Ribonuclease H2 subunit A
SAMHD1: Sterile alpha motif and histidine-aspartic acid domain-containing protein 1
SASP: Senescence-associated secretory phenotype
SENP7: Sentrin/SUMO-specific protease 7
SERCA: Sarcoplasmic endoplasmic reticulum calcium ATPase
ssDNA: Single-stranded DNA
STIM1: Stromal interaction molecule 1
STIM1: Stromal interaction molecule 1
STING: Stimulator of interferon genes
SUMO: Small ubiquitin-like modifier
TBK1: TANK-binding kinase 1
TF: Transcription factor
TFAM: Transcription factor A, mitochondrial
TIS: Therapy-induced senescence
TLRs: Toll-like receptors
TREX1: Three prime repair exonuclease 1
TRIM56: Tripartite motif-containing protein 56
TTLL6: Tubulin tyrosine ligase-like 6
ULK1: UNC-51-like kinase
UPR: Unfolded protein response
VDAC1: Voltage-dependent anion channel 1
WIPI2: WD-repeatPtdIns(3) P effector protein)
Y: Tyrosine
